# Health Data Processes: A Framework for Analyzing and Discussing Efficient Use and Reuse of Health Data With a Focus on Patient-Reported Outcome Measures

**DOI:** 10.2196/12412

**Published:** 2019-05-21

**Authors:** Niels Henrik Ingvar Hjollund, José Maria Valderas, Derek Kyte, Melanie Jane Calvert

**Affiliations:** 1 Occupational Medicine, University Research Clinic AmbuFlex/WestChronic Aarhus University Herning Denmark; 2 Department of Clinical Epidemiology Aarhus University Hospital Aarhus Denmark; 3 University of Exeter Collaboration for Academic Primary Care, Health Services & Policy Research Group National Institute for Health Research Collaboration for Leadership in Applied Health Research and Care (South West Peninsula) University of Exeter Exeter United Kingdom; 4 Centre for Patient Reported Outcomes Research Institute of Applied Health Research University of Birmingham, Edgbaston Birmingham United Kingdom; 5 National Institute for Health Research Birmingham Biomedical Research Centre and National Institute for Health Research Surgical Reconstruction and Microbiology Research Centre University of Birmingham, Edgbaston Birmingham United Kingdom

**Keywords:** medical informatics, patient-reported outcome, patient-physician relationship, data collection

## Abstract

The collection and use of patient health data are central to any kind of activity in the health care system. These data may be produced during routine clinical processes or obtained directly from the patient using patient-reported outcome (PRO) measures. Although efficiency and other reasons justify data availability for a range of potentially relevant uses, these data are nearly always collected for a single specific purpose. The health care literature reflects this narrow scope, and there is limited literature on the joint use of health data for daily clinical use, clinical research, surveillance, and administrative purposes. The aim of this paper is to provide a framework for discussing the efficient use of health data with a specific focus on the role of PRO measures. PRO data may be used at an individual patient level to inform patient care or shared decision making and to tailor care to individual needs or group-level needs as a complement to health record data, such as that on mortality and readmission, in order to inform service delivery and measure the real-world effectiveness of treatment. PRO measures may be used either for their own sake, to provide valuable information from the patient perspective, or as a proxy for clinical data that would otherwise not be feasible to collect. We introduce a framework to analyze any health care activity that involves health data. The framework consists of four data processes (patient identification, data collection, data aggregation and data use), further structured into two dichotomous dimensions in each data process (level: group vs patient; timeframe: ad hoc vs systematic). This framework is used to analyze various health activities with respect to joint use of data, considering the technical, legal, organizational, and logistical challenges that characterize each data process. Finally, we propose a model for joint use of health data with data collected during follow-up as a base. Demands for health data will continue to increase, which will further add to the need for the concerted use and reuse of PRO data for parallel purposes. Repeated and uncoordinated PRO data collection for the same patient for different purposes results in misuse of resources for the patient and the health care system as well as reduced response rates owing to questionnaire fatigue. PRO data can be routinely collected both at the hospital (from inpatients as well as outpatients) and outside of hospital settings; in primary or social care settings; or in the patient’s home, provided the health informatics infrastructure is in place. In the future, clinical settings are likely to be a prominent source of PRO data; however, we are also likely to see increased remote collection of PRO data by patients in their own home (telePRO). Data collection for research and quality surveillance will have to adapt to this circumstance and adopt complementary data capture methods that take advantage of the utility of PRO data collected during daily clinical practice. The European Union’s regulation with respect to the protection of personal data—General Data Protection Regulation—imposes severe restrictions on the use of health data for parallel purposes, and steps should be taken to alleviate the consequences while still protecting personal data against misuse.

## Introduction

Health information is central to all types of activities in the health care system, all of which involve collecting, analyzing, or using health information [1]. Securing personal data against misuse is the background for several legal initiatives, for instance, the implementation of the European Union’s General Data Protection Regulation (GDPR) [2]. One key element of this regulation is the principle that personal data collected for one purpose may not be immediately transferred and used for other purposes. However, while misuse of personal data poses a severe ethical problem, so does waste and duplicate collection of the same data from the same patients due to legal, organizational, and technical dysfunction. In addition, from the patient’s perspective, duplicate collection of data may be unnecessarily burdensome and time consuming, and the possibilities and advantages of alternative uses of health data should therefore be considered. We have discussed the patient’s perspective of joint use in more detail elsewhere [3].

Health information may be generated as an integrated part of health care activities, such as biochemical variables or entries in hospitals’ electronic health record (EHR) system, or it can obtained directly from the patient. The latter is the case for patient-reported outcome (PRO) measures, which have been defined by the US Food and Drug Administration as measurements “of any aspect of a patient’s health that comes directly from the patient, without interpretation of the patient’s responses by a physician or anyone else” [4]. This definition emphasizes the standardization of PRO data as opposed to unstructured clinician-reported summaries of patient history contained in the notes in patients’ health records.

The evaluation of treatment outcomes for each individual patient is typically captured by a combination of biological data, physical examination, and communication with the patient. However, evaluations of treatment outcomes at a group level (defined geographically, administratively, epidemiologically, or at the facility level) often focus solely on mortality; readmission; and, if available, data such as medicine use and other use of health services. Although these outcomes are undeniably important, they may fail to fully capture treatment outcomes. PRO measures can be used to complement such data as a primary or an additional distal outcome, or even serve as a proxy for an unmeasured clinical variable when collection of the latter is not feasible [5].

Health informatics aims to respond to the increasing demands of systematic collection and processing of data to inform individual patient care, service improvements, and precision medicine. A lot of effort and resources are expended on collecting, processing, storing, and retrieving health information (both PRO measures and other clinical measurements) such as in hospitals’ EHR systems. In parallel, an increasing number of research projects and initiatives independently collect health information for their own specific objectives. Although health informatics, as a discipline, engages with stakeholders from a wide range of professional backgrounds, roles, and interests, it mostly does so with a focus on one specific single application (clinical practice, clinical research, administrative purposes, surveillance, or computer science), as evidenced in textbooks and the relevant literature [1]. As a consequence, there is limited literature on the joint use of health information for several purposes.

Technical, legal, organizational, and other types of obstacles to the availability of data for multiple purposes result in inefficient use of resources among patients and clinicians as well as in the health care system and society. Where there is no additional benefit from repeating a measurement, the same health information should be collected only once. A typical example would be laboratory tests, which may be performed by the family doctor before referral to the hospital, but which may be repeated, in many circumstances, unnecessarily, once the patient arrives at the hospital. Similarly, clinicians frequently struggle to retrieve measurements needed for maintaining quality registers even though they may already be recorded in the EHR system. Similarly, PRO measurements may be repeatedly and independently collected in parallel for different purposes, such as clinical management, quality surveillance, and research projects. This may not just mean a waste of resources and an unnecessary burden to patients, but may also have implications for data quality, as response fatigue may lead to reduced response rates.

To qualify the discussion of efficient use of health data, we need a common language usable for all stakeholders, which does not exist. The aim of this paper is to propose a framework for analysis of use and reuse of health information, with a specific focus on the role of PRO measures in order to initiate and facilitate a more precise discussion.

## Definitions

There is no consensus on the method to define health information and health data. All definitions rely on the concepts of *information* (facts about a situation, person, event, etc [6]) and the *organized* property of data. Data have been defined accordingly in various ways such as (1) “any organized information collected by a researcher” [7], (2) “information or knowledge represented or coded in some form suitable for better usage or processing” [8], or (3) “information, especially facts and numbers, collected to be examined and considered and used to help decision making” [6]. The first definition focuses on the collection process and excludes purposes other than research, while the second one relies on data structure only. The third one identifies three processes relevant to the health data: collection, examination, and use; it furthermore acknowledges that the nature of data is preserved even if they are only stored and not used, at least not immediately.

**Figure 1:**
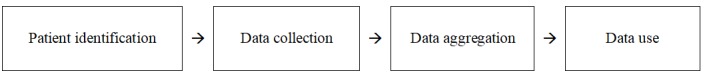
The four data processes in the lifespan of patient-related health data. Patient identification process: Identification of patient(s) from whom data are to be collected. Data collection process: The actual collection of health data including logistic procedures. Data aggregation process: Management and organization of collected data for the data use process. Data use process: Use of the health data for the purpose of the specified activity. Each process may be repeated or may take place simultaneously with the previous process. Further information is provided in Textbox 1.

In this paper, we use the third definition and differentiate three data processes: *data collection*, *data aggregation*, and *data use*. As patients are the unit of observation for health data, we need to additionally consider a *patient-identification* process to define whose data will be collected. We will focus on persons who may have, or are under surveillance for, a health condition and use the term “patient” even though some may not have a medical diagnosis. A generic model for health data covering any patient-related health data *activity* is shown in Figure 1. Definitions are summarized in Textbox 1.

The *patient-* *identification* process corresponds to the definition of the patient or the group of patients that will be the ultimate source of data. The subsequent *data collection* process contains measurement methods for generating data for that patient or population of patients as well as logistic issues. In the *data aggregation* process, data are transferred, organized, and transformed to enable their subsequent use. Aggregation may include data logistic procedures like transmission, data reformatting, and data management procedures such as combining and merging with other data. The aggregation may be explicit during data management (eg, a specific data manager making the dataset ready for the researcher’s use) or implicit (eg, such as a clinical summary based on patient data in an emergency room). In the *data use* process, the aim for the actual health data activity is fulfilled (eg, publish the results or a clinical decision of a treatment plan). Any pair of consecutive data processes may be repeated and make take place simultaneously.

Two dimensions can be recognized across all four data processes: *level* and *timeframe*. Level may be either the *individual* patient or a defined patient *group* level (eg, patients admitted to a hospital department or patients with a specific health condition). Timeframe considers the scope of the health data activity and may be either *ad hoc* or part of a *systematic* planned process. Examples are provided below.

**Figure 2:**
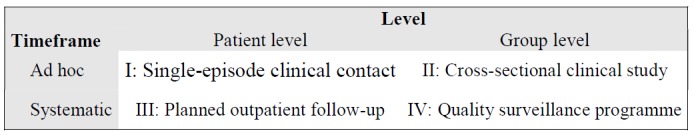
Examples of basic health data activities, where the same cell is used in all the four health data processes. I: The patient makes an appointment, and during the consultation, data are collected and aggregated to make a clinical decision and treatment plan (all four data processes ad hoc at the patient level). II: The target population is identified and data are collected, managed, analyzed, and published (all data processes ad hoc at a group level). III: An inpatient is discharged and referred for continuous planned outpatient follow-up and data are collected during follow-up, aggregated at each visit, and used at the visit (all data processes are systematic at the patient level) IV: Patient groups are identified repeatedly (eg, once a year) based on some criteria and data are collected, managed, and analyzed/reported (all data processes take place systematic at a group level).

Based on these dimensions, 2 × 2 tables with four cells may be constructed for each of the four processes. Four basic health data activities may be defined, where the same cell is used in all the four data processes in Figure 1. Figure 2 shows such examples.

In most heath data activities, different cells are applied in the four health data processes, and these patterns will be analyzed to highlight their properties and differences.

**Definitions of health data terms.**

Health data: Health information about individual patients

Health data activity: An activity with a health-related aim that uses or produces health data

Health data processes: Any health data activity includes four processes:

- Patient identification process: Identification of patient(s) from whom data are to be collected
- Data collection process: The collection of health data
- Data aggregation process: Transfer or organization of health data in a way that enables data use
- Data use process: Use of health data for the purpose of a specified health data activity

Timeframe: The timeframe of a health data process:

- Systematic: A planned or repeated health data process
- Ad hoc: A nonplanned process

Level: The level of a health data process:

- Patient level: The individual patient level
- Group level: A level with patients grouped according to some defined criteria

## Basic Health Data Activities

Each data process of basic health data activities (Figure 2) is described below and displayed in Table 1. A description of the contents of each process is shown in the Multimedia Appendix 1.

### The Single-Episode Clinical Contact Activity

The patient makes an appointment with the general practitioner. During the consultation, data are collected by medical history and physical examination. These data and a general view of the patient and his/her resources are aggregated by the general practitioner into a conclusion and used for a clinical decision. Another single-episode example is an emergency room visit. The timeframe for all the four data processes listed in Figure 1 is *ad hoc,* and all take place at the *patient level*. The content of the data collection process may include systematic methods such as standardized blood tests or use of a specific validated questionnaire, in which case, the timeframe is *ad hoc*.

**Table 1:** Examples of basic and complex health data activities divided by level and timeframe. In basic health data activities, all four processes are in the same level/timeframe cell.

Health data process		Patient identification				Data collection				Data aggregation				Data use			
		Patient		Group		Patient		Group		Patient		Group		Patient		Group	
		Ad hoc	Sys^a^	Ad hoc	Sys	Ad hoc	Sys	Ad hoc	Sys	Ad hoc	Sys	Ad hoc	Sys	Ad hoc	Sys	Ad hoc	Sys
**With basic data process patterns**																	
	Single-episode clinical contact	✓^b^				✓^b^				✓^b^				✓^b^			
	Planned patient follow-up		✓^b^				✓^b^				✓^b^				✓^b^		
	Clinical research (cross-sectional)			✓^b^				✓^b^				✓^b^				✓^b^	
	Quality surveillance program				✓^b^				✓^b^				✓^b^				✓^b^
**With complex data process patterns**																	
	Clinical research (cohort)			✓^b^					✓^b^			✓^b^				✓^b^	
	Clinical guideline			✓^b^	✓				✓^b^			✓^b^	✓	✓^b^			
	Individual prognosis forecast			✓^b^	✓				✓^b^	✓^b^		✓^b^		✓^b^			
	Screening program				✓^b^				✓^b^		✓^b^			✓^b^			
	Disease surveillance	✓	✓		✓^b^		✓^b^						✓^b^				✓^b^
	Health care error surveillance	✓^b^				✓^b^				✓			✓^b^	✓			✓^b^
	Primary health care, traditional	✓^b^	✓			✓^b^				✓^b^				✓^b^	✓		
	Primary health care, new trend	✓^b^	✓		✓	✓^b^	✓		✓	✓^b^	✓		✓	✓^b^	✓		✓

^a^Sys: Systematic or repeated data process.

^b^The most frequently applied data processes.

### The Planned Outpatient Follow-Up Activity

Many patients with a chronic disease have systematic follow-ups in an outpatient clinic. The patients are referred to outpatient follow-up in a systematic manner based on written formal or local informal guidelines. The data needed for the outpatient consultation (eg, medical history, laboratory tests, PRO measures, and physical examination) are collected, aggregated, and used at the patient level in relation to each visit. The timeframe for all the four data processes is *systematic* and takes place at the *patient level*.

### The Cross-Sectional Clinical Research Activity

In a cross-sectional study, the group of patients is defined once, the data are collected and analyzed once, and the results based on condensed data are published once. Another example is a registry-based study. The timeframe for all four data processes is *ad hoc* and takes place at a *group level*.

### The Quality Surveillance Program Activity

The quality surveillance program is an ongoing activity, where at a defined timepoint, (eg, once a year), data are sampled and subsequently analyzed with respect to differences between departments and hospitals. Reports are published and used for optimizing quality of care or to inform the patient’s choice of health care provider. The timeframe for all four data processes is *systematic* with predefined intervals and take place at a *group level*.

## Complex Health Data Activities

Although the abovementioned activities apply data processes in the same cell in all four data processes, most health data activities combine different cells. Table 1 (lower part) shows examples of such activities. The list is not comprehensive but represents examples of the possible combinations of data process patterns.

### The Longitudinal Clinical Research Activity

The patient-identification process—the recruitment (eg, for a clinical trial)—takes place once, or in the case of an open cohort study, systematically over a long inclusion period. Data are collected *systematically* over time according to a defined study protocol. Aggregation (data management and analysis) and use (publication) take place only once. All data processes are *systematic* and take place at a *group level*. As discussed later, these data can be used for a range of other purposes.

### The Clinical Guideline Activity

Clinical guidelines are based on meta-analyses of clinical trials and longitudinal studies collected at a *group level*. Data are aggregated to inform the guidelines and are published and implemented once or at regular intervals. The use of clinical guidelines is, however, most often *ad hoc* at the individual *patient level* when clinical decisions about diagnostic procedures and treatment are made at the “bedside” together with that specific patient. In many countries, the traditional *ad hoc* use of guidelines is being replaced by quality programs or pay-per performance systems with the purpose of implementing the guidelines for *all* relevant patients. This will move the data use process from *ad hoc* to *systematic* at the *patient level*.

### The Individual Prognosis Activity

Like treatment guidelines, prognostic indicators rely on information collected at the *group level*. Prognostic forecasts are used at the individual *patient level* and use the experiences of cohorts of patients to provide information on individual prognosis. Prognostic information may also be used as decision support together with the patient, for example, to choose between two treatments. Two approaches that differ with respect to the aggregation process—model based or data based—may be distinguished. In the model-based approach, the data are aggregated once at the *group level* and published as, for example, an equation based on regression coefficients, while in the data-based approach, data are aggregated from the cohort data each time the prognosis is asked for, and the prognosis for a subgroup with characteristics similar to the patient is selected and displayed [9]. Traditionally, prognosis has been expressed in terms of clinical outcomes (survival, readmission etc), but PRO measures may be used to include outcomes such as symptom burden and functioning.

### The Screening Program Activity

In a population screening program, citizens or patients to be invited are identified *systematically* based on risk factors such as age; gender; and at times, disease-specific risk factors. The data collection takes place at the *group level*, but the aggregation and use processes occur at the individual *patient level*, since each screening-positive citizen is referred and further diagnosed and treated individually.

### The Disease Surveillance Activity

Registers for monitoring diseases have been known since the middle of the 19th century, when the first-known registry was established with the purpose of monitoring leprosy at the population level [10]. Relevant patients are preferably identified based on diagnosis codes in existing registers, but a number of disease registers still rely on reports from the individual clinician, as do etiological registers like worker’s compensation registers. Secondary collection of data (eg, histologic type of cancer or treatment) is organized *systematically* at the *patient level*.

### The Health Care Error Surveillance Activity

Health care error is, by nature, an *ad hoc* event at the *patient level*. In surveillance, patients are identified and data are collected *ad hoc* at the *patient level* and aggregated to statistics and reports at the *group level* (eg, hospital, department, or physician). In case of a serious error, the data may also be used at the individual level as a basis for audit, compensation, or even legal action.

### Activities in Primary Health Care

Traditionally, all four data processes in primary health care have been *ad hoc* at the *patient level*, except for *systematic, group-level* programs like vaccination, pregnancy, and maternal care as well as some mandatory reporting of summary statistics to medical authorities. However, in some countries, primary care activities go from *ad hoc* to *systematic* *ally* framed processes at the *group level* (eg, chronic care programs), where the general practitioner is expected to identify patients with certain profiles, and primary health care quality surveillance programs based on *group-level* aggregation of clinical data are also being implemented.

## Patient-Reported Outcome Measures in the Data Collection Process

PRO-based health data are not essentially different from other sources of information with respect to the data processes of identification, aggregation, and use, but the data collection process has a number of features that are specific for PRO measures. First, without PRO measures, health data on symptoms and functioning are difficult to collect systematically and will be limited to observations and unsystematic clinician-reported subjective summaries of patient history, which frequently underestimate patient problems [11]. Second, PROs are often the only way to collect data from a patient at home (telePRO). A number of telehealth projects have tried to collect data from home with various hi-tech methods with impact limited to few specific diseases, whereas *telePRO* has shown robustness and been used in a range of chronic diseases [12]. In the following section, PRO-specific aspects of activities listed in Table 1 will be highlighted.

### Patient-Reported Outcome in Patient-Level Activities

**Figure 3:**
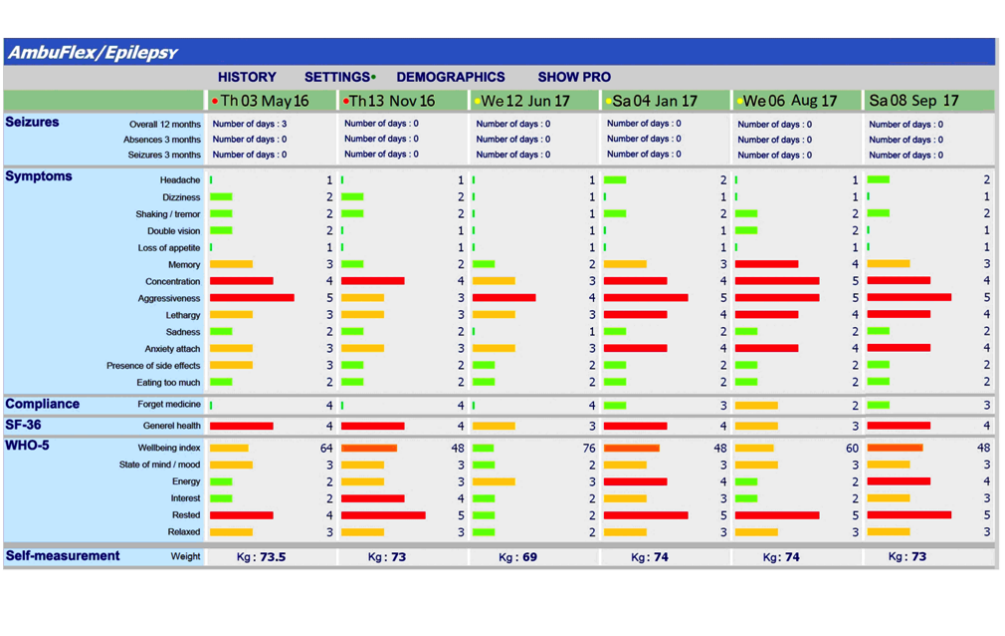
Longitudinal overview of patient-reported outcome and self-reported measurements in outpatient follow-up (translated from Danish) [13].

Paper-based patient questionnaires have been used in the clinical setting for decades to support the communication between the patient and physician. The PRO data are aggregated and used during the consultation as a tool to screen for a priori defined, critically important symptoms (red flags) and to prioritize issues based on the patient’s preferences. This use of PRO measures has increased with the introduction of Web-based questionnaires, patient kiosks in the waiting area, etc. The effects on the consultation processes have been reviewed elsewhere [13-15]. During patient follow-up, PRO data are collected in connection with each scheduled visit and used to support a longitudinal overview of symptoms and functions over time and to provide real-time warnings of deterioration aimed at facilitating a prompt response from the care team (Figure 3). If patients complete the PRO remotely online, usually at home (*telePRO*), this information may be used as the base for demand-driven outpatient follow-up without prebooked visits, where disease-relevant PRO questionnaires filled in at home at fixed intervals are aggregated by a disease-specific algorithm that semiautomatically decides whether there is a need or wish for an outpatient visit [12]. This may solve the paradox that outpatient clinics may be drowning in patients even though a substantial part of the visits turn out to be unnecessary from both the patient’s and clinician’s point of view [16-18]. In Denmark, this principle has been implemented in chronic and malignant diseases including asthma, chronic obstructive pulmonary diseases, epilepsy, sleep apnea, prostatic cancer, and chemotherapy for a number of malignant diseases [19]. A national implementation of the principle is underway in Denmark for selected diagnostic groups.

### Patient-Reported Outcome in Group-Level Activities

The clinical research, quality surveillance, clinical guideline, and individual prognosis activities (Table 1) rely on similar data and will be discussed together. PRO data collection has been applied for decades in clinical research based on the belief that outcomes cannot be evaluated on the basis of clinical measures only. Ideally, most group-based activities need longitudinal data with a long follow-up period, often beyond the time span of outpatient follow-up. Due to the increasing use of PRO measures for clinical purposes, isolated collection becomes problematic because the patient is often reluctant to answer more than one questionnaire, especially when the relevance is not clear and questions across measures overlap, leading to repeated questions with similar content [20].

The demand for data by the health care system will undoubtedly increase in the future for all described activities, with the cross-sectional study as a possible exception. Most of the listed data-demanding activities focus on longitudinal data, and the following discussion will focus on this and the role of PRO measures in a longitudinal follow-up.

## Multiple Use of Data Collected as Part of Clinical Follow-Up

Of the four data processes, the data collection process is the main challenge with respect to costs as well as logistics. To reduce costs and workload among patients and clinicians, it is essential to focus on joint efforts of data collection with subsequent use in other health data activities. To some extent, this is already happening (eg, clinical research based on clinical quality databases).

The basic example of longitudinal activity is the patient follow-up, where information on the course of treatment, symptoms, and effect of the intervention is monitored, and, if necessary, treatment is adjusted. This activity is *systematic* at the *patient level*, and data are already stored for documentation purposes and may therefore potentially be reused in other activities. A schematic overview of principles in joint efforts where data collected from patient follow-up are used in other activities is shown in Table 2. For the activities listed in Table 1, data aggregation and data use are unchanged; only the processes in the alternative patient identification and data collection processes differ.

**Figure 4:**
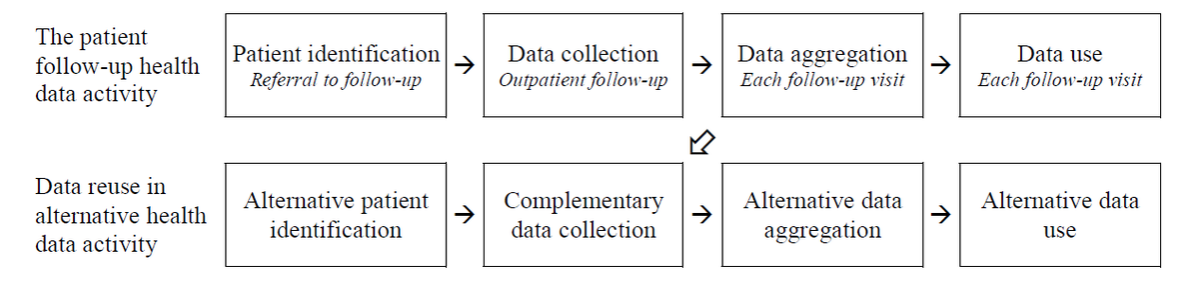
Joint use of health data based on data collected during patient follow-up. The oblique arrow indicates identification of missing patients, observations, and variables for alternative use.

A model for joint use of health data based on data collected during patient follow-up with secondary identification of missing patients, observations, and variables for the alternative use is shown in Figure 4. The methods used for identification of missing patients, observations, and variables for the alternative use (Figure 4) depend on the timeframe of the alternative use. If the *ad hoc* method is used, data are exported to an external system where the completeness is analyzed with record linkage methods similar to those used in normal registry-based research, followed by additional *ad hoc* data collection. If the alternative use is to take place repeatedly in a systematic manner, this detection of missing data should preferably take place with online access to the environment in which the clinical data reside. In the Central Denmark Region, a central data warehouse has been established, which now contains clinical information on medication, diagnoses, and procedures, and more information is being collected [21]. These data are available for use in quality-improvement projects, but according to the GDPR, the use of data for research requires the patient to provide explicit permission, which reduces the possibility for joint use significantly.

**Table 2:** Examples of joint use of health data based on reuse of data routinely collected during patient follow-up with alternative patient identification, complementary data collection, alternative aggregations, and uses of data.

Examples	Patient identification				Data collection				Data aggregation				Data use			
			
	Patient		Group		Patient		Group		Patient		Group		Patient		Group	
	Ad hoc	Sys^a^	Ad hoc	Sys	Ad hoc	Sys	Ad hoc	Sys	Ad hoc	Sys	Ad hoc	Sys	Ad hoc	Sys	Ad hoc	Sys
Clinical practice		✓^b^			Basis^c^	Basis				✓				✓		
Quality surveillance				✓		Reuse^d^	Comp^e^					✓				✓
Clinical research			✓			Reuse	Comp				✓				✓	
Individual prognosis			✓			Reuse	Reuse		✓		✓		✓			

^a^Sys: Systematic or repeated data process.

^b^All check marks indicate unchanged activity-specific processes (see Table 1).

^c^Basis: The routine collected follow-up data are the base for alternative uses.

^d^Reuse: Direct reuse of data collected in the cell above.

^e^Comp: Complementary data collection.

*Group-level ad hoc* procedures are applied to identify missing patients, observations, and variables, with subsequent complementary data collection. In quality surveillance programs, identification takes place as a *systematic group-level* process, but data collection should rely mainly on data collected as part of normal clinical activity. In clinical research, the identification of patients to be included will be *ad hoc*, based on the specific research protocol, while data collection should rely on data collected as part of normal clinical activity as the primary source of data, supplemented with additional *ad hoc* data collection, when supplementary outcome assessment is needed. Individual prognosis will most often rely solely on data collection in research activities, but as the only other activity, data-based individual prognosis may entirely rely on data collected as part of clinical follow-up, given that the data are available for instantaneous on-the-fly aggregation and use [9].

## Patient-Reported Outcome Data Collection Supplemental to Data Collected During Follow-up

Different health data activities may have different data demands with respect to timing and PRO content, which makes supplemental PRO data collection necessary. PRO collection in clinical practice does not cover the whole population of patients, since some patients may not attend follow-up and some patients may, for one reason or another, not complete a questionnaire. For clinical use, response rates over 90% are obtainable in *telePRO* [12,22]. For the traditional use of clinical PRO measures, typically collected in the waiting room area, PRO data are obtained only from patients who turn up at follow-up visits, and the response rate is dependent on local commitment, influenced by local population characteristics and configuration of the system (user interface, accessibility and compatibility) and integrated within the existing EHR, clinical pathways, and workflow.

Clinical data collection stops when the patient follow-up ends. For reuse in other activities, it may be necessary to apply supplemental data collection. It is possible to incorporate supplementary research items into PRO questionnaires used for clinical purposes as well as to extend the follow-up period longer than clinically relevant for data use in the activities of clinical research, quality surveillance, and individual prognosis. With respect to content, PRO questionnaires used for a clinical purpose may not be appropriate for other activities, but with respect to domains to be covered, the common set between the activities is often substantial. For example, the European Organisation for Research and Treatment of Cancer scales created for use at *group level* are often usable for *telePRO* [23] when supplemented with a few items, most importantly, the patient’s preference for contact with the health care provider.

High response rates are crucial for PRO data collection in any health data activity, and PRO data collection in clinical practices often has higher response rates than PRO data collected for use at *group level* [22], where the response rate is dependent on local coordination and commitment [24]. Response rates are dependent on how relevant the data appear to the patient, and clinical use seems nearest to the patient. PRO data collected in clinical practice may therefore yield higher quality of data than traditional surveillance studies. A known problem when PRO data are collected at a *group level* is what to do with alarming answers, the so-called PRO “alerts” such as high depression scores or signs of suicidal ideation [25]. This is feasible to deal with in a clinical setting but is very difficult when collecting data only for *group-level* use. While supplemental data collection of clinical data may be troublesome and expensive due to several reasons such as extra follow-up visits, PRO data collection processes may be centralized and automated if the relevant infrastructure is available [22].

## Challenges in Reuse of Patient-Reported Outcome Data Collected During Follow-up

In order to achieve the anticipated potential of joint use of PRO data, some critical challenges should be addressed. The psychometric requirements may vary depending on the specific use (eg, level of reliability and sensitivity to change), but other requirements for the data collection process, such as high response rate, low attrition rate, and high data completeness, remain essentially the same regardless of the activity and type of data. All activities must meet challenges in terms of data collection logistics and management, and the demands for data security are typically also identical. Supplemental data collection requires close cooperation between PRO activities with real-time access to data, which raises some issues. The challenges are divided into three types.

### Legal Challenges

Legislation issues have a bearing on all four data processes; therefore, the legal framework has to be precisely specified before any data collection can begin. Activities with *systematic* data collection may typically benefit from permanent permission from national data protection agencies, while *ad hoc* projects must apply for permission for a specified period. The fundamental problem is that all approvals are only valid for the specific activity (eg, quality surveillance or clinical research), which means that data cannot be used for other activities. The implementation of the European Union’s regulation with respect to protection of personal data—GDPR [2]—will make it even more difficult to use data for other purposes without a specific consent from each patient. This will have a serious impact on joint use unless health data are given a differentiated treatment, such that the requirements for confidentiality can be maintained and individual approval can be collected in an efficient way (eg, through some form of umbrella approval process). For *group-level* use, analyses of personal data may be performed on a remote server where the researcher may upload a dataset and merge it with personal data using a unique personal identifier. The researcher has access to only aggregated data such as tables and outputs from statistical analyses [26]. Such a method of accessing personal data is available in Denmark and the Netherlands, but for now, few health data such as those on diagnoses and procedures are available for merging.

### Technical Challenges

The principle of supplemental identification and data collection described above presupposes real-time access to relevant patient databases in the patient-identification process and in most cases, in the data collection process. Apart from that, there are substantial technical issues related to the aggregation process. Data may be collected and stored, but not available for the relevant alternative aggregation. A typical example is the quality surveillance activity, where the needed data may already exist in the patient’s EHR, but an automated process of extracting and transporting data is not possible due to inadequate and incompatible information technology systems or a lack of relevant expertise. PRO data may already be collected but stored in a different system or format. A possible solution to the latter is proposed by the international Health Level Seven standards for transfer of clinical and administrative data between software apps used by various health care providers [27]. A special Health Level Seven section for PRO measures has recently been adopted.

### Challenges Related to Content and Timing of Data Collection

The need for valid, reliable, and responsive measurement scales is common for data for any health data activity. For PRO data collected for making individual clinical decisions, measurement error is of particular importance. Although scales that have acceptable psychometric properties at the patient level will normally also perform well at a *group level*, the opposite is not true and the desirable content and length of a PRO questionnaire are likely to differ between *group-level* and *patient-level* activities. In routine patient follow-up visits, short instruments are often preferred and procedures that the clinician finds irrelevant for the actual patient may not be collected as prescribed (eg, a comprehensive time-consuming test of performance in a patient who has clinically completely recovered or a depression score in a patient who is clinically obviously not depressed). A possible solution to these contradictive interests may be application of item banks and computer-adaptive testing, which can achieve high reliability with the lowest-possible administration burden [28]. Timing of data collection poses another challenge for joint use, and the optimal timing of data collection may differ between activities. Quality surveillance and clinical research may prefer that data collection follow a fixed scheduled in compliance with a protocol, while outpatient clinical practice is focused on the practical arrangement of follow-up, and visits often have to be postponed for various reasons. Although from a clinician’s perspective, it might be acceptable that patients who are doing well cancel their appointments, this may result in devastating selective attrition in *group-level* activities. From a resource point of view and the patient’s perspective, a patient who does not need or want clinical attention should not go to follow-up visits just to deliver data for other purposes. A rational approach for addressing these problems with missing data for the alternative activity could be a supplemental real-time identification of patients with missing data combined with collection of PRO data on proxy variables.

## Conclusions

We have introduced a model for health data with four data processes, each dividable with respect to timeframe and group level, which distinguishes properties relevant to the discussion of joint use across different purposes and supports consideration of the associated organizational and technological challenges. Based on this, we propose a model for joint use of health data, with data collected during follow-up as the backbone. In the future, clinical settings will be a prominent source of PRO data and data collection for research and quality surveillance will have to adapt to this circumstance and design ways of complementary data collection as and when necessary. Demands for health data will continue to increase, which will further add to the need for the concerted use and reuse of PRO data for parallel purposes due to financial, logistical, and ethical reasons. A number of legal, technical, and organization challenges must be addressed.

The risk of patients’ information being accessed and used by people for whom it was not initially intended is real. For example, the use of health data by private insurance companies might restrict access to health coverage for vulnerable patients and those with a precondition. Additionally, access to private medical information by law enforcement agencies could be a risk for individuals and society. However, the current legal restriction on the joint use of health data imposed by the GDPR makes no distinction between these misuses and the uses described in this paper. Steps should be taken to alleviate the current legal restriction on the joint use of health data imposed by the GDPR while still protecting patient data against misuse.

## Appendix

Multimedia Appendix 1 Examples of basic health data activities.

## Abbreviations

EHR: electronic health record
GDPR: General Data Protection Regulation
PRO: patient-reported outcome
