# Supplementary material for: Health Data Processes: A Framework for Analyzing and Discussing Efficient Use and Reuse of Health Data With a Focus on Patient-Reported Outcome Measures
Source: J Med Internet Res. 2019 May 21;21(5):e12412. doi: 10.2196/12412 (PMC6547770; doi:10.2196/12412)
Supplement: Multimedia Appendix 1 [file jmir_v21i5e12412_app1.pdf]

Data process 1: Patient identification

Timeframe of patient identification	Level of patient identification	
	Patient level	*Group level*
Ad hoc	I: Patient makes an appointment	II: Cross-sectional sampling
Systematic	III: Referral to outpatient follow-up	IV: Panel identification

Data process 2: Data collection

Timeframe of data collection	Level of data collection	
	Patient level	*Group level*
Ad hoc	I: Consultation	II: Cross-sectional data collection
Systematic	III: Outpatient follow-up	IV: Cohort follow-up

Data process 3: Data aggregation

Timeframe of data aggregation	Level of data aggregation	
	Patient level	*Group level*
Ad hoc	I: Summary, diagnosis	II: Data management
Systematic	III: Outpatient follow-up	IV: Data management

Data process 4: Data use

Timeframe of data use	Level of data use	
	Patient level	*Group level*
Ad hoc	I: Treatment plan	II: Hypothesis test & publication
Systematic	III: Treatment adjustment	IV: Case mix analyses & quality report

Figure. Examples of basic health data activities where the same cell is used an all the four health data processes. The four data processes of health data divided by level and timeframe
I: Single episode clinical contact, II: Cross-sectional clinical study, III: Planned outpatient follow-up and IV: Quality surveillance programme (cf. Fig 2).
